# Umbilical cord arterial blood lactate dehydrogenase and pH as predictors of perinatal outcome in high-risk term pregnancies: A cohort study

**DOI:** 10.34763/jmotherandchild.20222601.d-22-00004

**Published:** 2022-07-20

**Authors:** Naina Kumar, Ashu Yadav

**Affiliations:** Department of Obstetrics and Gynecology, All India Institute of Medical Sciences, Bibinagar, Hyderabad, Telangana, India; Department of Obstetrics and Gynecology, Maharishi Markandeshwar Institute of Medical Sciences and Research, Ambala, Haryana, India

**Keywords:** Birth Asphyxia, Encephalopathy, Neonate, Pregnancy Umbilical cord

## Abstract

**Background:**

Birth asphyxia is a common cause of perinatal morbidity, mortality.

**Objective:**

To compare the efficacy of umbilical cord arterial blood lactate dehydrogenase (LDH) and pH as predictors of neonatal outcome in high-risk term pregnancies using receiver operating characteristic (ROC) curves.

**Material and methods:**

Present retrospective cohort study was conducted in the rural tertiary centre of Northern India over two years (January 2017–December 2018). Neonates delivered to 300 term (≥37 – ≤42 weeks) high-risk antenatal women were enrolled after fulfilling inclusion criteria. Immediately after delivery of a newborn by any mode, the segment of the umbilical cord (10 cm) was double clamped, cut, and arterial blood samples were taken for LDH and pH and were compared with neonatal outcome. Statistical analysis was done using SPSS 22.0 software.

**Results:**

For all 300 neonates mean ± SD values of cord blood LDH and pH were 545.19 ± 391.93 U/L and 7.13 ± 0.15, respectively. High cord blood lactate and low pH values were significantly associated with adverse neonatal outcomes including neonatal resuscitation, NICU admission, complications and early neonatal deaths (p=0.000). The sensitivity, specificity and negative predictive value of cord blood LDH in the prediction of neonatal death was 100.00%, 53.17%, 100%, and pH was 93.75%, 53.17%, 99.34%, respectively.

**Conclusion:**

Cord blood lactate and pH help in the early prediction of neonatal outcomes, but cord blood lactate is a better predictor.

## Introduction

All over the world, around 130 million infants are born annually, out of which 4 million die in their first 28 days of life [1]. According to recent data, around 2.5 million children had died during the first month of life in 2018 worldwide, accounting for approximately 7,000 neonatal deaths each day [2]. A major proportion (three-quarters) of these neonatal deaths occur in the first week of birth [3,4], with one-third occurring on the first day of life [2]. The major causes for such high perinatal mortality are infections (36%), preterm births (28%) and birth asphyxia (23%), together accounting for 87% of all neonatal deaths worldwide [3, 4, 5]. Furthermore, it was observed that 99% of these neonatal deaths occur in developing countries where perinatal asphyxia is a major cause, accounting for 23% of these deaths [4]. India alone is responsible for around one-fifth of global live births and more than 25% of neonatal deaths. An estimated 0.75 million neonatal deaths were observed in India in 2013, the highest for any country all over the world [6], with perinatal asphyxia contributing to almost 20% of these deaths [7]. Moreover, around one million children, who survive birth asphyxia, suffer from chronic neuro-developmental morbidities, including cerebral palsy, mental retardation and learning disabilities in the latter part of their life [8].

The exact definition of birth asphyxia is still imprecise but can be defined as ‘failure to initiate and sustain breathing at birth leading to hypoxia of various organs [9,10]’. The American College of Obstetrics and Gynaecology in 2002 recommended criteria for diagnosis of intrapartum asphyxia; including four essential and five additional criteria [11]. The essential criteria included metabolic acidosis (pH < 7.0 and base deficit ≥ 12 mmol/L) in the umbilical artery, moderate or severe encephalopathy, cerebral palsy and exclusion of other aetiologies. The five additional criteria are sentinel event, abrupt changes in foetal heart rate, Apgar score ≤ 3 beyond 5 min, multi-system failure within 72 hours of life and early imaging evidence.

Umbilical cord blood analysis may be used for the early prediction of hypoxia-related damages and metabolic acidosis in the foetus. The commonly used methods are cord arterial blood pH and lactate dehydrogenase (LDH) level estimation. Hypoxia causes organ damage, and the liver is one such organ that is highly vulnerable to hypoxic damage, resulting in an early, abrupt and transient increase in aminotransferases, alkaline phosphatase and LDH levels [12]. The foetal lactate, especially during hypoxia, is cleared from the placenta. Umbilical artery lactate levels can thus serve as an efficient and accurate technique for diagnosing foetal hypoxia [13,14]. Furthermore, LDH levels correlate well with disease severity, such as asphyxia, respiratory distress, necrotizing enterocolitis and hypoxic ischemic encephalopathy (HIE) [15].

Hence, the present study was conducted to know the role of umbilical cord arterial blood pH and LDH levels in the early prediction of adverse perinatal outcomes in term neonates of high-risk pregnancy, to reduce the overall burden of perinatal morbidity and mortality associated with birth asphyxia.

## Material and Methods

**Study Design**: A retrospective cohort study

**Study Population**: Present study was conducted in the department of Obstetrics and Gynaecology of a rural tertiary care centre of Northern India over 2 years (January 2017 to December 2018).

**Inclusion Criteria**: All neonates delivered to antenatal women at gestation ≥37 weeks, with singleton pregnancy with high-risk factors like anaemia, hypertensive disorders of pregnancy, previous history of caesarean section, malpresentation, thyroid disorders, gestational diabetes mellitus, seizure disorders, Rh incompatibility, intrauterine growth restriction with or without oligohydramnios, polyhydramnios, prelabour rupture of membranes (PROM), maternal infections, intrahepatic cholestasis of pregnancy.

**Exclusion criteria**: Antenatal women with preterm or post-term delivery (<37 or >42 weeks), low-risk or normal pregnancy, intrauterine foetal death, congenitally malformed foetus, multi-foetal gestation, neonates delivered to mothers who had received magnesium sulfate within 4 hours before delivery or who had impending cesarean scar dehiscence or rupture and those refusing to participate were excluded from the study.

**Sampling Procedure**: All the study participants were selected using consecutive sampling techniques until the required duration of the study was reached.

**Study Sample**: Three hundred (300) neonates delivered to women with a high-risk pregnancy at term in the department of Obstetrics and Gynaecology of a rural tertiary care centre of Northern India were enrolled as study subjects.

**Ethical Issues**: The present retrospective cohort study was conducted after Institutional Ethical Clearance (IEC number: 1070) and informed written consent from the participants in their vernacular language.

### Data Collection

The socio-demographic features like age, gestation, parity, high-risk factors and mode of delivery were recorded on a structured data collection sheet by trained nursing staff. A detailed medical and family history of all the participants was taken to ensure that they fulfil the inclusion criteria for the study. This was followed by a thorough physical examination of every subject that was recorded. The blood pressure of all the subjects at admission to the labour ward and after 2 hours of rest was carefully noted. In addition to routine investigations and obstetric ultrasounds, organ-specific blood investigations were sent depending on the history and presentation of participants like liver, kidney and thyroid function tests. Around 10 ml of midstream urine sample was collected in a clean container for urinary protein analysis using dipsticks. Results of all the investigations were recorded on a performed proforma, and a diagnosis of the high-risk factor was made accordingly.

Immediately after vaginal/caesarean delivery the umbilical cord was doubly clamped and cut at two places keeping a segment of 10–12 cm. The umbilical cord arterial blood samples were then drawn from the segment and collected anaerobically in a pre-heparinized insulin syringe, which was sent directly for pH estimation at 37°C using a blood gas analyser and around 0.5 ml of sample was put in a lithium heparin tube for LDH analysis. Neonatal gender, Apgar scores at 1- and 5-minutes, birth weight, need for resuscitation, NICU admission, early neonatal complications and overall neonatal outcome were recorded by the paediatrician on a final datasheet.

### Laboratory Assessments

Siemens arterial blood gas analyser machine was used for estimation of umbilical cord arterial blood pH immediately after birth. In term neonates, the normal reference value for cord arterial blood pH is 7.24 ± 0.07 [16]. The arterial blood sample (0.5 ml) in lithium heparin test tube for analysis of LDH levels was transferred to the biochemistry department within a few minutes of delivery, where the total LDH activity was measured using the standard Deutsche Gesellschaft fur Klinische Chemie (DGKC) method. In this method, the test tubes containing cord blood samples were first centrifuged followed by their analysis using the Erba Mannheim XL System (ERBA diagnostics Mannheim GmbH, Germany). Two reagents, R1 and R2, were used in this system; R1 contains Tris buffer (pH 7.5) 100 mmol/l and Pyruvate 2.0 mmol/l and R2 has NADH 1.66 mmol/l. The principal reaction used for estimation of LDH activity was: Pyruvate + NADH + H+ → Lactate + NAD+. Lactate dehydrogenase reduces pyruvate to lactate, which in turn causes oxidation of reduced nicotinamide adenine dinucleotide (NADH) to NAD. This rate of oxidation of NADH is directly proportional to cord blood LDH activity, estimated by the decreased rate in absorbance at 340 nm as NAD is produced. The normal reference value for cord blood LDH in a term newborn is <612 U/L [17].

### Statistical Analysis

Statistical analysis of data was performed using Statistical Package for Social Sciences (SPSS) software version 22.0. The continuous variables were compared using unpaired t-test/Mann-Whitney U-test, whereas the ANOVA/Kruskal Wallis test was used for comparison of two or more than two groups. For qualitative variables, Chi-Square test or Fisher’s exact test was used. Categorical variables were expressed as frequencies and percentages, wherever needed. A p-value of <0.05 was considered statistically significant. The receiver operating characteristic (ROC) curve was used to demonstrate the diagnostic accuracy (sensitivity and specificity) of umbilical cord arterial blood LDH levels and pH in prediction of early neonatal mortality.

## Results

### Socio-demographic outcomes

Of the total 300 neonates delivered to high-risk pregnant women, 55.7% delivered between >37 and ≤39 weeks, 40% between >39 and ≤41 weeks, and 4.3% between >41 and ≤42 weeks. Of all, 164(54.7%) were male neonates and 136(45.3%) females. The average age of all the antenatal women was 27.43 years, with the majority belonging to the 21–30 years of age group (77.3%). The majority of cases had multiple risk factors (59.3%). The common high-risk factors observed were hypertensive disorders of pregnancy (29.3%), previous caesarean scar (17.7%), anaemia (17.3%), thyroid function disorders (15.0%), Rh incompatibility (13.7%), liquor abnormalities (11.0%), intrauterine foetal growth restriction (10.7%), intrahepatic cholestasis of pregnancy (9.7%), maternal infections (9.3%), gestational diabetes mellitus (8.7%), malpresentation (8.3%), PROM (8.0%) and bad obstetric history (6.7%). No significant correlation was observed between type of maternal high-risk factors and neonatal Apgar scores, umbilical cord arterial blood pH and LDH values (p>0.05). The most common mode of delivery was vaginal (44.3%) followed by lower segment caesarean section (39%) and instrumental delivery (15.7%). The various participants characteristics are depicted in Table 1.

**Table 1:** Participant Characteristics

Parameter	Number (n)	Percentage
**MATERNAL AGE (years)**		
19–20	7	2.3%
21–30	232	77.3%
31–40	61	22.3%
**GRAVIDITY**		
G1	62	20.7%
G2	91	30.3%
G3	86	28.7%
G4	38	12.7%
≥ G5	23	7.7%
**PARITY**		
0	92	30.7%
1	113	37.7%
2	81	27.0%
≥3	14	4.7%
**GESTATIONAL AGE (WEEKS)**		
>37– ≤39	167	55.7%
>39– ≤41	40	40%
>41– ≤42	13	4.3%
**MODE OF DELIVERY**		
Normal vaginal delivery	133	44.3%
Emergency lower-segment caesarean section	75	25.0%
Elective lower-segment caesarean section	42	14.0%
Instrumental delivery	47	15.7%
Breech-assisted vaginal delivery	3	1.0%
**NEONATAL GENDER**		
Male	164	54.7%
Female	136	45.3%
**NEONATAL BIRTH WEIGHT (KG)**		
≥1.5–≤2.5	34	11.3%
>2.5–≤3.5	253	84.3%
≥3.5	13	4.3%
**NEONATAL OUTCOME**		
Survived	284	94.7%
Early neonatal death	16	5.3%

A significant association was observed between the mode of delivery and Apgar scores, cord arterial blood pH and LDH levels as depicted in Table 2.

**Table 2:** Association between mode of delivery, Apgar scores, cord blood pH and LDH levels

Parameter	Normal vaginal delivery	Emergency LSCS*	Elective LSCS^*^	Instrumental delivery	Assisted breech delivery	Total	Chi- square value	P value
**1-min Apgar score**								
<7	14(15.2%)	41(44.6%)	4(4.3%)	31(33.7%)	2(2.2%)	92(30.7%)	83.88	0.000
>7	119(57.2%)	34(16.3%)	38(18.3%)	16(7.7%)	1(0.5%)	208(69.3%)		
**5-min Apgar score**								
<7	0(0%)	5(62.5%)	0(0%)	2(25%)	1(12.5%)	8(2.7%)	20.75	0.000
>7	133(45.5%)	70(24.0%)	42(14.4%)	45(15.4%)	2(0.7%)	292(97.3%)		
**Cord arterial blood pH**								
<7.2	37(25.0%)	51(34.5%)	16(10.8%)	42(28.4%)	2(1.4%)	148(49.3%)	67.69	0.000
≥7.2	96(63.2%)	24(15.8%)	26(17.1%)	5(3.3%)	1(0.7%)	152(50.7%)		
**Cord arterial blood LDH**								
<612U/L	112(59.6%)	30(16.0%)	35(18.6%)	9(4.8%)	2(1.1%)	188(62.7%)	88.59	0.000
≥612U/L	21(18.8%)	45(40.2%)	7(6.3%)	38(33.9%)	1(0.9%)	112(37.3%)		

^*^LSCS: Lower segment caesarean section

Furthermore, neonates delivered by emergency caesarean section for non-reassuring foetal heart rate had significantly lower cord blood pH values (chi-square value= 7.48; p=0.006) and high LDH levels (chi-square value= 6.49; p=0.01) as compared to those undergoing caesarean section for other indications. A total of 61(20.3%) cases had meconium-stained liquor with or without features of non-reassuring foetal heart rate. The cord blood pH was significantly lower (chi square=35.983; p=0.000), and the LDH levels were significantly higher (chi square=47.45; p=0.000) in neonates delivered to mothers with meconium-stained liquor.

## Neonatal Outcome

Of the total 300 neonates delivered, 92(30.7%) and eight (2.7%) had one and five-minute Apgar scores <7, respectively. The umbilical cord arterial blood pH was <7.24 in 148 (49.3%) neonates (p>0.05), and the cord blood LDH levels were >612U/L in 112(37.3%) neonates (p>0.05). The mean ± SD values for 1-minute Apgar scores in neonates with cord blood pH <7.24 and LDH levels ≥612U/L were 7.10 ± 0.62 (p=0.000) and 6.04 ± 0.95 (p=0.000), respectively. The neonatal gender and birth weight had no significant effect on cord blood pH and LDH values. A total of 75(25.0%) neonates required resuscitation at birth, 115(38.3%) NICU admission, 90(30.0%) developed early neonatal complications and 16(5.3%) had early neonatal deaths. Twelve (4.0%) neonates developed HIE as a consequence of birth asphyxia, of which two (16.7%) had grade 1 HIE with mean ± SD cord blood pH of 6.87 ± 0.24 and LDH levels as 909.80 ± 108.9, eight (66.7%) had HIE 2 with cord blood pH of 6.91 ± 0.18 and LDH levels as 1160.06 ± 539.1, and two (16.7%) had HIE 3 with cord blood pH of 6.80 ± 0.0 and LDH levels as 1875.90 ± 381.9, respectively. The relation between overall neonatal outcome and Apgar scores, cord blood pH and LDH levels are depicted in Table 3.

**Table 3:** Association between umbilical cord arterial blood pH, lactate dehydrogenase levels and overall neonatal outcome

Parameter		Neonatal resuscitation		NICU admission		Early neonatal complications		Early neonatal death	
		Yes	No	Yes	No	Yes	No	Yes	No
**Cord blood pH**	<7.2	66(44.6% )	82(55.4%)	100(67.6 %)	48(32.4% )	79(53.4%)	69(46.6%)	15(10.1 %)	133(89.9% )
	≥7.2	9(5.9%)	143(94.1 %)	15(9.9%)	137(90.1 %)	11(7.2%)	141(92.8 %)	1(0.7%)	151(99.3% )
Chi-square value		59.82		105.61		76.02		13.34	
p-value		0.000		0.000		0.000		0.000	
**Cord blood**	<612	6(3.2%)	182(96.8 %)	14(7.4%)	174(92.6 %)	9(4.8%)	179(95.2 %)	0(0%)	188(100%)
**LDH (U/L)**	≥612	69(61.6% )	43(38.4%)	101(90.2 %)	11(9.8%)	81(72.3%)	31(27.7%)	16(14.3 %)	96(85.7%)
Chi-square value		127.74		203.22		152.43		28.37	
p-value		0.000		0.000		0.000		0.000	

**Figure 1:**
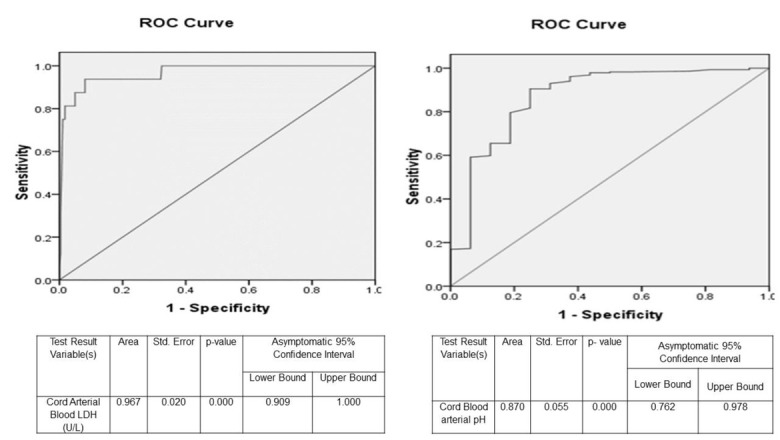
Receiver operating characteristic (ROC) curves plotting the sensitivity and specificity of the umbilical cord arterial blood lactate dehydrogenase and pH values in the prediction of early neonatal mortality

It was observed that cord blood LDH levels had significantly better sensitivity (100% vs. 93.75%), positive (14.3% vs. 10.1%), negative predictive values (100% vs. 99.34%) and overall accuracy rate (68.0% vs. 55.3%) as compared to cord blood pH values in the prediction of neonatal outcome. Figure 1 depicts the ROC curves plotting the sensitivity and specificity of the umbilical cord arterial blood lactate dehydrogenase and pH values in the prediction of early neonatal mortality.

The area under the curve (AUC) for cord blood lactate is 0.967 and for cord blood arterial pH is 0.870, indicating that cord blood lactate is an outstanding test for prediction of neonatal mortality as compared to cord blood arterial pH.

## Discussion

The present retrospective cohort study was conducted on 300 neonates delivered to term high-risk antenatal women with the mean gestational age of 37.84 ± 5.69 weeks. The most common high-risk factors observed were hypertensive disorders of pregnancy (29.3%) and previous caesarean scar pregnancy (17.7%). The majority of the neonates were delivered vaginally followed by lower segment caesarean section (emergency and elective LSCS) with majority having birth weight between >2.5 and ≤3.5 kgs.

In the present study, it was observed that the mode of delivery was significantly associated with risk of birth asphyxia, low one- and five-minute Apgar scores, low cord blood pH and high LDH values. Furthermore, the association was highly significant for emergency LSCS done for non-reassuring foetal heart rate and meconium-stained liquor. A recent study reported a significant association between high cord arterial blood LDH levels and primigravidity, meconium-stained liquor and with administration of oxytocin. Similar to our study they also observed no significant association between cord arterial blood lactate levels and neonatal birth weight [18]. In our study no significant association was found between neonatal sex and cord blood pH and LDH levels. Other studies have reported a significant relation between male sex and high cord blood lactate levels in neonates [18, 19]. Another recent cross-sectional study reported that neonates delivered with meconium-stained liquor had significantly lower Apgar score, lower cord blood pH and high umbilical cord blood lactate levels. They also concluded that cord blood lactate has a high sensitivity and specificity for distinguishing thick and thin meconium forms in amniotic acid and, hence, the severity of meconium aspiration syndrome (MAS) and need for ventilation in newborns with this syndrome [20]. A similar study conducted on neonates delivered to mothers with meconium-stained amniotic fluid observed a significantly lower cord blood pH values and high cord blood lactate levels and base deficits in neonates with MAS as compared to those without MAS [21]. Similar to our findings, a study reported that the neonates delivered by emergency caesarean section had significantly higher cord arterial blood LDH levels as compared to those delivered vaginally. They also concluded that neonates delivered by elective LSCS had significantly lower cord blood LDH levels [22]. Similar results were reported by a recent retrospective study, which concluded that the lowest values of cord blood lactate were observed in neonates delivered by elective caesarean section, and the highest values were observed in those delivered by instrumental vaginal deliveries [23]. Another study concluded that the lowest umbilical cord blood pH levels were found in neonates delivered instrumentally (vacuum or forceps), and the highest pH among neonates delivered by elective LSCS [24]. A study has reported that the cord blood LDH values were significantly elevated in newborns delivered with meconium-stained amniotic fluid and the degree of rising, strongly associated with the amount of hypoxia [25]. Similar results were reported by many other studies also [26,27].

In our study, a significant correlation was observed between low Apgar scores, need for neonatal resuscitation, NICU admission, and development of neonatal complication with low cord arterial blood pH values and high LDH levels. It was observed that neonates having cord blood LDH values >612U/L had to mean Apgar scores at 1 and 5 minutes as 6.04 ± 0.95 and 7.90 ± 1.05, respectively, and neonates with cord blood pH value <7.2 had mean Apgar as 6.29 ± 0.86 and 8.14 ± 0.98, respectively. This was similar to the results of a study, which observed that 87.5% of neonates with Apgar score ≤7 at 1 minute had elevated cord blood LDH, and only 68.7% of neonates with Apgar score ≤7 at 5 minutes had elevated cord blood LDH levels. Furthermore, they observed that the specificity of serum lactate (97.7%) and umbilical artery pH (95.97%) was almost similar in neonates with Apgar score ≤7 at 1 min, but the sensitivity of serum lactate (23.14%) and cord pH (31.4%) was less in neonates with Apgar score ≤7 at 1 minute [28]. Similar findings were also reported by another study that compared neonatal Apgar scores at birth and umbilical cord blood lactate levels and creatinine in neonatal having perinatal asphyxia and concluded that cord blood lactate assay helped in better prediction of severity of foetal anoxia [29]. A recent study concluded that the cord blood pH at birth in combination with Apgar scores is a good predictor of severity of birth asphyxia and early neonatal outcome. They also concluded that the Apgar scores and cord blood pH were inversely related to duration and severity of birth asphyxia [30]. Other similar studies have also observed that elevated cord arterial blood lactate levels were a better predictor of NICU admission as compared to cord blood pH, further suggesting the role of LDH in decision making in the early neonatal period [26,31].

In the present study, 100% of the expired neonates had raised cord blood LDH levels (>612U/L), while 93.8% had umbilical cord arterial pH values < 7.2 (p=0.000). Hence, though both cord blood pH and LDH levels were found to be good predictors of neonatal complications and outcome, cord blood LDH was observed to have significantly higher sensitivity 100% (95% CI: 79.41% to 100%), specificity of 53.17% (95% CI: 47.18% to 59.04%), positive 14.3% (95% CI: 12.41% to 16.40%) and negative predictive values (100%) and overall better accuracy (68% vs. 53.35%), respectively, in the prediction of neonatal outcome. This was similar to the results of a recent study, which concluded that umbilical cord blood lactate levels are a better predictor of adverse neonatal outcome than umbilical cord pH [32]. Another recent study also reported that the sensitivity of LDH was 94.3%, specificity 87.5%, positive predictive value (PPV) 92.7%, negative predictive value (NPV) 90.1% and the diagnostic accuracy was 91.8% for birth asphyxia [33]. A similar study found that cord blood LDH has a sensitivity of 94%, specificity of 90%, and positive and negative predictive values of 90.38% and 93.75%, respectively, in the prediction of neonatal birth asphyxia [34]. Furthermore, a study reported that LDH levels of ≥810 IU/L can accurately predict asphyxia and adverse neurodevelopmental outcomes in newborns with a specificity of 95.65%, sensitivity of 54.55% and positive likelihood ratio of 12.54 [35]. Other studies have also reported that cord blood lactate levels can be used as a potential screening test for neonatal acidosis [36,37].

## Conclusion

Hence, it can be concluded that both umbilical cord arterial blood pH and LDH can help in the prediction of overall neonatal outcome including Apgar scores at birth, neonatal resuscitation, NICU admission rate, neonatal complications and neonatal death. It was further observed that cord blood LDH was a better predictor of NICU admission, neonatal complications including the severity of HIE, and neonatal death as compared to cord blood pH with higher sensitivity and specificity as it reflects minor distress during the delivery of the neonates even in the absence of acidosis at birth and/or poor Apgar scores. Therefore, umbilical cord arterial blood pH and LDH can be used for early prediction and intervention, so as to reduce the overall neonatal morbidity and mortality.

## Key Points

- High cord blood lactate and low pH values were significantly associated with adverse neonatal outcomes in high-risk term pregnancies.
- The umbilical cord blood LDH levels had better sensitivity (100% vs 93.75%), positive (14.3% vs. 10.1%), negative predictive values (100% vs. 99.34%) and overall accuracy rate (68.0% vs. 55.3%) as compared to cord blood pH in the prediction of neonatal outcome in high-risk term pregnancies.
- The AUC in ROC curve for cord blood lactate was 0.970, indicating that it is an outstanding test for prediction of early neonatal mortality in high-risk term pregnancies.
- The cord blood pH was significantly lower (chi square=35.983; p=0.000) and LDH levels were significantly higher (chi square=47.45; p=0.000) in neonates delivered to high-risk mothers with meconium-stained liquor.
- No significant correlation was observed among the type of maternal high-risk factors and neonatal Apgar scores, umbilical cord arterial blood pH and LDH values.
- The neonatal gender and birth weight had no significant effect on cord blood pH and LDH values.

## Figure Legends

**Figure 1**. Receiver operating characteristic (ROC) curves plotting the sensitivity and specificity of the umbilical cord arterial blood lactate dehydrogenase and pH values in the prediction of early neonatal mortality
